# Red cell distribution width to platelet ratio predicts liver fibrosis in patients with autoimmune hepatitis

**DOI:** 10.1097/MD.0000000000021408

**Published:** 2020-08-21

**Authors:** Huali Wang, Jian Wang, Juan Xia, Xiaomin Yan, Yanhong Feng, Lin Li, Jun Chen, Duxian Liu, Weimao Ding, Yongfeng Yang, Rui Huang, Chao Wu

**Affiliations:** a Department of Hepatology, Nanjing Second Hospital, Nanjing University of Chinese Medicine; b Department of Infectious Diseases; c Department of Pathology, Nanjing Drum Tower Hospital, The Affiliated Hospital of Nanjing University Medical School; d Department of Pathology, Nanjing Second Hospital, Nanjing University of Chinese Medicine, Nanjing; e Department of Hepatology, Huai’an No. 4 People's Hospital, Huai’an, Jiangsu, China.

**Keywords:** autoimmune hepatitis, liver fibrosis, noninvasive tests, red cell distribution width

## Abstract

Noninvasive tests for the assessment of liver fibrosis are highly needed for the management of patients with autoimmune hepatitis (AIH). We aimed to investigate the accuracy of red cell distribution width to platelet ratio (RPR) in predicting liver fibrosis in AIH patients. One hundred nineteen AIH patients who underwent liver biopsy were enrolled. Liver fibrosis stage was diagnosed using the Scheuer scoring system. The diagnostic accuracy was evaluated by the area under the receiver operating characteristic curve (AUROC). RPR values in AIH patients with S2-S4 (0.10, interquartile range [IQR] 0.08–0.15), S3-S4 (0.10, IQR 0.09–0.14), and S4 (0.14, IQR 0.09–0.19) were significantly higher than patients with S0-S1 (0.07, IQR 0.06–0.08, *P* < .001), S0-S2 (0.08, IQR 0.06–0.12, *P* = .025) and S0-S3 (0.09, IQR 0.07–0.13, *P* = .014), respectively. The RPR was positively correlated with fibrosis stages (*r* = 0.412, *P* < .001), while aspartate transaminase to platelet ratio index (APRI) and fibrosis-4 score (FIB-4) were not significantly associated with fibrosis stages in AIH patients. The AUROCs of RPR in identifying significant fibrosis (S2-S4), advanced fibrosis (S3-S4), and cirrhosis (S4) were 0.780 (95% confidence interval [CI] 0.696–0.865), 0.639 (95% CI 0.530–0.748), and 0.724 (95% CI 0.570–0.878), respectively. The AUROCs of RPR were significantly higher than APRI and FIB-4 in diagnosing significant fibrosis, advanced fibrosis, and cirrhosis. Our study demonstrates that the RPR is a simple predictor of liver fibrosis and is superior to APRI and FIB-4 in identifying liver fibrosis in AIH patients.

## 1 Introduction

Autoimmune hepatitis (AIH) is an immune-mediated chronic inflammatory liver disease, which is classically characterized by elevated serum transaminase and immunoglobulin G levels, hypergammaglobulinemia, specific autoantibodies production, and liver interface hepatitis on pathological examination.^[1,2]^ Chronic liver inflammation can result in liver fibrosis, cirrhosis, and hepatic carcinoma in AIH.^[3]^ Given the lack of specific diagnostic markers for AIH patients, majority of patients already have significant fibrosis or even cirrhosis when they are first diagnosed.^[3]^ Evaluating the stages of liver fibrosis is essential for choosing treatment strategies and estimating long-term prognosis for AIH patients.^[4]^

Liver biopsy (LB) is the gold standard to assess disease activity and liver fibrosis in AIH patients.^[4,5]^ However, LB is not an optimal method for evaluating liver fibrosis due to its invasiveness, high cost, sampling errors, and observer discrepancy.^[6,7]^ In addition, it is difficult to observe the dynamical changes of liver fibrosis by LB. Therefore, noninvasive tests (NITs) for assessing liver fibrosis were developed in the past years. Transient elastography (TE) is a promising method with high accuracy for evaluating liver fibrosis in chronic viral hepatitis.^[8–10]^ However, the predicting accuracy of TE in AIH patients is controversial, since elevated alanine aminotransferase (ALT) levels may influence the accuracy of TE in detecting early stages of fibrosis.^[11]^ In addition, high cost of equipment limits the clinical use of TE in resource-limited settings.^[12]^ Several NITs based on clinical parameters for assessing liver fibrosis have been established, including aspartate transaminase (AST) to platelet (PLT) ratio index (APRI) and the fibrosis-4 score (FIB-4). APRI and FIB-4 were initially proposed to assess liver fibrosis with relatively high accuracy in patients with chronic hepatitis C (CHC).^[13,14]^ APRI and FIB-4 are also recommended to assess significant fibrosis and cirrhosis in both chronic hepatitis B (CHB) and CHC patients by the World Health Organization.^[12,15]^ However, several studies reported that the predicting performances of these 2 NITs in AIH patients were not satisfied.^[16,17]^

A simpler and easy-to-calculate NIT, red cell distribution width (RDW) to PLT ratio (RPR), was developed to assess liver fibrosis and cirrhosis for CHB patients.^[18]^ The area under the receiver operating characteristic curves (AUROCs) of RPR were 0.825 and 0.884 for diagnosing significant fibrosis and cirrhosis in CHB patients, which were superior to the FIB-4 and APRI.^[18]^ In other liver diseases, RPR also has an excellent performance for predicting liver fibrosis.^[19]^ Wang et al reported that RPR had a higher accuracy than APRI and FIB-4 in identifying significant fibrosis in patients with primary biliary cirrhosis (PBC).^[19]^ However, whether RPR can be used to predict fibrosis stages in AIH patients remains unclear. In the present study, we analyzed the diagnostic accuracy of RPR for significant liver fibrosis, advanced liver fibrosis, and liver cirrhosis in AIH patients. Furthermore, we compared the predicting values of RPR with APRI and FIB-4 for liver fibrosis stages.

## 2 Methods

### 2.1 Patients

Between July 2016 and June 2019, a total of 127 consecutive AIH patients from Nanjing Drum Tower Hospital, The Second Hospital of Nanjing and Huai’an No. 4 People's Hospital, who underwent LB were enrolled in the present study. One hundred four (81.9%) of the patients were female and the median age of patients were 53.0 (interquartile range [IQR] 46.0, 60.0) years. AIH patients were diagnosed according to the practice guidelines of the American Association for the Study of Liver Diseases.^[4]^ None of patients received immunosuppressive therapy before LB. Patients with the following conditions were excluded from the study:

(1) combined with other liver diseases, such as viral hepatitis, nonalcoholic fatty liver disease (NAFLD), alcoholic liver disease, PBC, and metabolic liver disease;
(2) co-existence of hepatic carcinoma or other malignant tumor;
(3) severe cardiac, respiratory, renal, hematological, and psychiatric diseases.

Among the 127 patients, 5 patients who were combined with CHB, 2 patients with CHC, and 1 patient with insufficient data were excluded. Finally, 119 AIH patients were included for analysis in this study.

All patients provided written informed consent for the LB, and this study was performed according to the ethics principles of the Declaration of Helsinki and approved by the Ethics Committees of Nanjing Drum Tower Hospital, The Second Hospital of Nanjing and Huai’an No. 4 People's Hospital.

### 2.2 LB and laboratory test

Ultrasound-guided LB was performed using a 16-gauge disposable needle. All liver specimens were scored by pathologists blinded to patient clinical characteristics. Liver fibrosis stages were evaluated according to the Scheuer scoring system.^[20]^ Liver fibrosis was classified into the following 5 stages: S0, no fibrosis; S1, portal fibrosis without septa; S2, portal fibrosis with rare septa; S3, numerous septa without cirrhosis; and S4, cirrhosis.^[20]^ S2-S4, S3-S4, and S4 are defined as significant liver fibrosis, advanced liver fibrosis, and liver cirrhosis, respectively. We retrospectively reviewed the medical records of the enrolled patients. Demographic and clinical characteristics were recorded within 1 week before LB, including age, sex, blood routine, biochemistry, and immunology tests.

### 2.3 Computational formula of NITs

The NITs used in the present study were as follows: APRI: (AST (U/L)/ULN of AST)/PLT count (10^9^/L) ×100^[13]^; FIB-4: (age (years) × AST (U/L))/ ((PLT count (10^9^/L) × (ALT (U/L))^1/2^)^[14]^; RPR: RDW (%)/PLT count (10^9^/L).^[18]^

### 2.4 Statistical analyses

Continuous variables were presented as the median (IQR) and were compared using the independent *t* test or Mann–Whitney *U* test. Categorical variables were expressed as percentages and were analyzed by Chi-square test. The correlation between NITs and liver fibrosis stage was determined using Spearman rank correlation test. Receiver operating characteristic (ROC) curves was performed to evaluate the predictive accuracy of different NITs. The AUROCs and 95% confidential interval (CI) of AUROC were calculated. Differences between the AUROCs were tested using the *z*-test. The cut-off values were determined by the Youden index which was the optimal combination of sensitivity and specificity. Differences were considered to be significant at a 2-tailed *P* < .05. All statistical analyses were carried out using the SPSS statistical software version 22.0 (SPSS Inc., Chicago, IL).

## 3 Results

### 3.1 Study population

A total of 119 AIH patients were included for the analysis. The characteristics of patients were shown in Table 1. The majority of patients were female (83.2%) and median age was 52.5 (IQR 44.5, 59.8) years old. The median levels of PLT, RDW, ALT, and immunoglobulin G were 156.5 (IQR 118.3, 196.5) × 10^9^/L, 13.7 (IQR 12.9, 15.6) %, 81.6 (IQR 40.0, 204.7) U/L, and 15.9 (IQR 12.8, 20.8) g/L, respectively. Eighty-two patients (68.9%) were positive for antinuclear antibody and 17 (14.3%) were positive for anti-smooth muscle antibody. The distributions of each liver fibrosis stage were as follows: S0, 4 (3.4%) patients; S1, 33 (27.7%) patients; S2, 48 (40.3%) patients; S3 22 (18.5%) patients; S4 12 (10.1%) patients. The median values of APRI, FIB-4, and RPR were 1.32 (IQR 0.56, 2.64), 2.69 (IQR 1.35, 5.10), and 0.09 (IQR 0.07, 0.13), respectively.

**Table 1:**
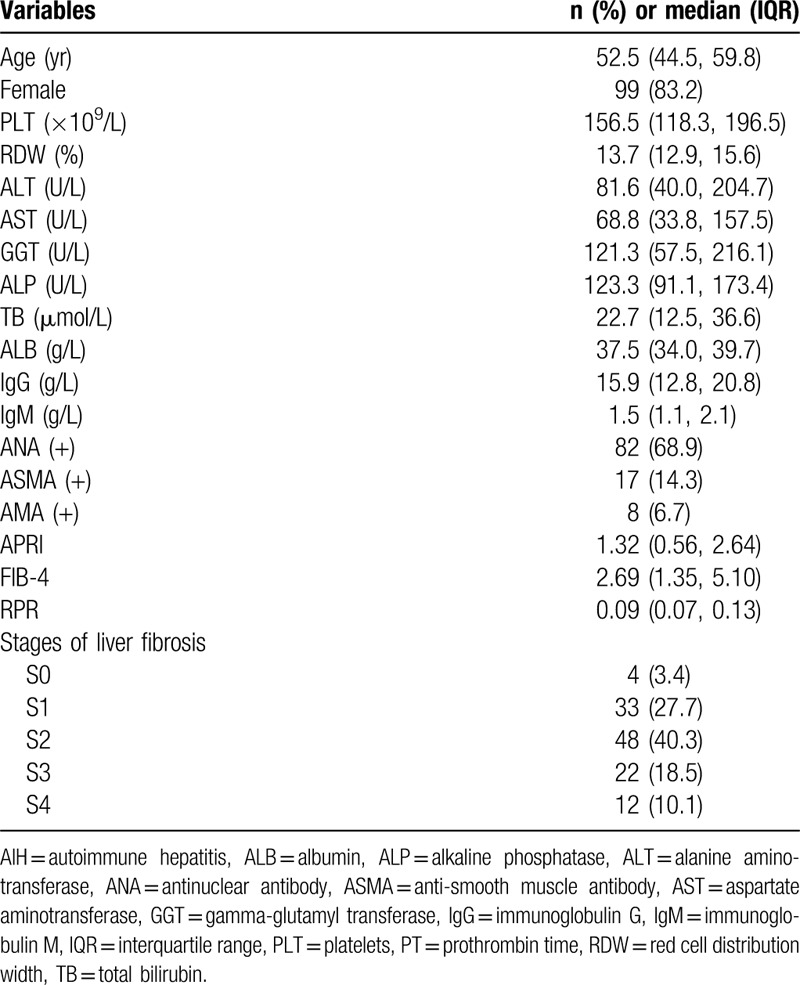
Baseline characteristics of study patients.

### 3.2 Comparisons of different NITs according to liver fibrosis stages

**Figure 1:**
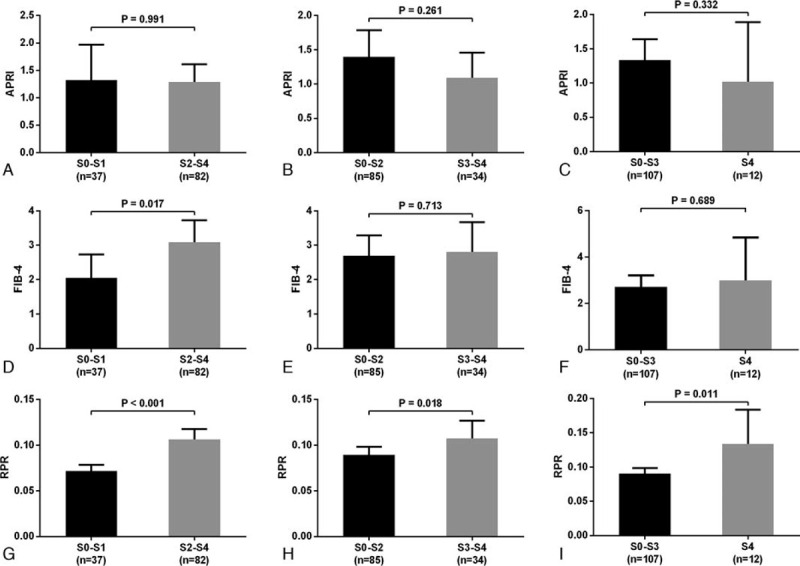
Comparisons of the APRI (A), FIB-4 (B), and RPR (C) levels according to different liver fibrosis stages in AIH patients. AIH = autoimmune hepatitis, APRI = aspartate transaminase to platelet ratio index, FIB-4 = fibrosis-4 score, RPR = red cell distribution width to platelet ratio.

**Figure 2:**
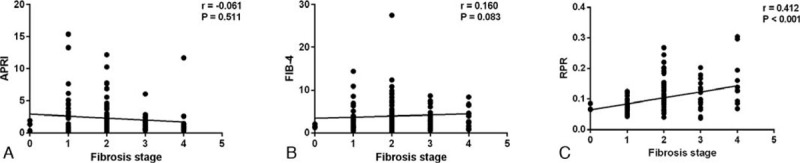
Correlations between different noninvasive tests and liver fibrosis stages.

The levels of APRI, FIB-4, and FIB-4 in different fibrosis stages were showed in Figure 1. The results showed that RPR values in patients with S2-S4 (0.10, IQR 0.08–0.15), S3-S4 (0.10, IQR 0.09–0.14), and S4 (0.14, IQR 0.09–0.19) were significantly higher than that of patients with S0-S1 (0.07, IQR 0.06–0.08, *P* < .001), S0-S2 (0.08, IQR 0.06–0.12, *P* = .018), and S0-S3 (0.09, IQR 0.07–0.13, *P* = .011), respectively. However, the values of APRI were not significant different between patients with S0-S1 (1.31, IQR 0.48–3.24) and S2-S4 (1.37, IQR 0.57–2.52, *P* = .991), S0-S2 (1.44, IQR 0.49–3.24) and S3-S4 (1.03, IQR 0.57–1.90, *P* = .261), S0-S3 (1.41, IQR 0.55–2.82) and S4 (0.89, IQR 0.58–1.40, *P* = .332). FIB-4 values in patients with S2-S4 (3.22, IQR 1.58–6.41) were significantly higher than that of patients with S0-S1 (1.83, IQR 1.22–3.76, *P* = .017), while the FIB-4 values were not significant different between patients with S0-S2 (2.62, IQR 1.26–5.18) and S3-S4 (2.99, IQR 1.53–5.06, *P* = .713), S0-S3 (2.69, IQR 1.29–5.10) and S4 (3.28, IQR 1.72–5.97, *P* = .689). The RPR was positively correlated with fibrosis stages (*r* = 0.412, *P* < .001), while the APRI (*r* = −0.061, *P* = .511) and FIB-4 (*r* = 160, *P* = .083) were not correlated with fibrosis stages (Fig. 2).

### 3.3 Comparisons of diagnostic accuracy between RPR and other NITs

**Figure 3:**
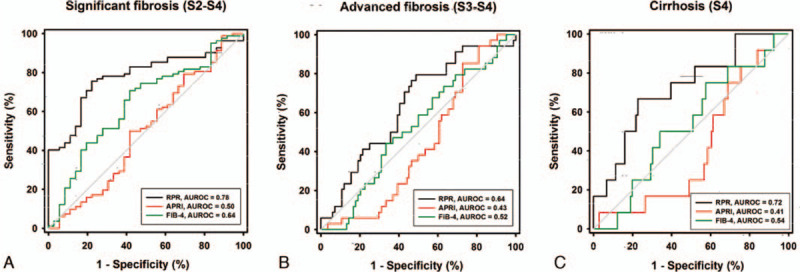
Receiver operating characteristic curve of different non-invasive tests for predicting significant liver fibrosis (A) advanced liver fibrosis (B), and liver cirrhosis (C) in AIH patients. AIH = autoimmune hepatitis.

The ROC curves were performed to evaluate the accuracy of RPR, APRI, and FIB-4 in identifying significant fibrosis, advanced fibrosis, and cirrhosis (Fig. 3). The AUROCs of RPR in predicting significant fibrosis, advanced fibrosis, and cirrhosis were 0.780 (95% CI 0.696–0.865), 0.639 (95% CI 0.530–0.748), and 0.724 (95% CI 0.570–0.878), respectively. The optimal cut-off values of RPR were 0.083, 0.084, and 0.127, respectively. The AUROCs of APRI in predicting significant fibrosis, advanced fibrosis, and cirrhosis were 0.499 (95% CI 0.379–0.620, *P* = .991), 0.434 (95% CI 0.329–0.539, *P* = .261) and 0.414 (95% CI 0.262–0.567, *P* = .332), and the AUROCs of FIB-4 in predicting significant fibrosis, advanced fibrosis, and liver cirrhosis were 0.639 (95% CI 0.529–0.748, *P* = .017), 0.522 (95% CI 0.410–0.634, *P* = .713) and 0.535 (95% CI 0.372–0.699, *P* = .689), respectively. In comparison, The AUROCs of RPR were significantly higher than APRI and FIB-4 in diagnosing significant fibrosis, advanced fibrosis, and liver cirrhosis (Table 2).

**Table 2:**
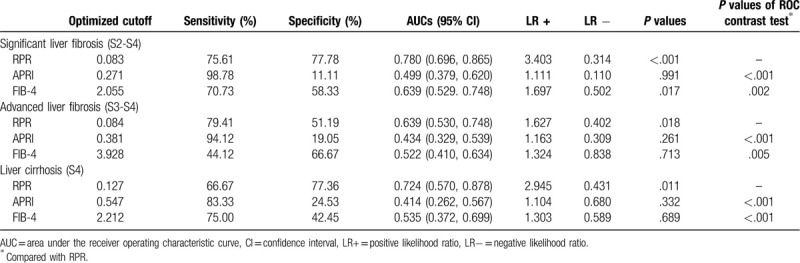
Diagnostic accuracy of different non-invasive tests for predicting liver fibrosis in patients with autoimmune hepatitis.

## 4 Discussion

NITs for accurately identifying liver fibrosis stages are highly needed for the clinical management of AIH. Although several NITs have been proposed to predict liver fibrosis with high accuracy in patients with viral hepatitis,^[13,18,21]^ the diagnosis performances of these NITs in AIH patients are still to be explored.

APRI and FIB-4 are 2 most widely used NITs of diagnosing liver fibrosis and are recommended by the World Health Organization guidelines to predict liver fibrosis in CHB and CHC patients in resource-limited settings.^[12,15,22–24]^ However, few studies have reported the performances of APRI and FIB-4 for predicting liver fibrosis in AIH patients. Yuan et al reported that the AUROCs of APRI and FIB-4 were 0.798 and 0.881 for predicting liver cirrhosis in AIH patients.^[25]^ Similar study was reported by Zeng et al which showed that APRI and FIB-4 could diagnose liver cirrhosis with moderate accuracy in AIH patients.^[16]^ However, these 2 studies only investigated the accuracy of APRI and FIB-4 in predicting liver cirrhosis in AIH patients. As compared to the diagnose liver cirrhosis, accurately evaluating early stages of liver fibrosis is more important for AIH patients.^[3]^ Moreover, the sample sizes are very small in these 2 studies.^[16,25]^ In the present study, we assessed the diagnostic performances of these 2 NITs for significant liver fibrosis, advanced liver fibrosis, and liver cirrhosis in AIH patients. However, our results suggested that APRI could not predict significant liver fibrosis, advanced liver fibrosis, and liver cirrhosis. FIB-4 could only identify significant liver fibrosis with a low AUROC of 0.639. Our study demonstrates that APRI and FIB-4 are not good NITs for staging liver fibrosis in AIH patients as in viral hepatitis.

In the present study, we further investigated the novel NIT, RPR, for staging liver fibrosis in AIH patients. The results revealed that the diagnostic performances of RPR for different liver fibrosis stages were significantly higher than that of APRI and FIB-4. RPR was initially established to estimate liver fibrosis in patients with CHB.^[18]^ RPR was demonstrated to predict significant fibrosis and cirrhosis in CHB patients with relatively high accuracy, which was superior to APRI and FIB-4.^[18]^ Since then, several studies have validated the performances of RPR for predicting liver fibrosis in chronic liver diseases.^[19,26–28]^ A retrospective study from Korean indicated that the diagnostic performance of RPR for predicting advanced liver fibrosis and cirrhosis was comparable to FIB-4 and superior to APRI in CHB patients.^[26]^ A systematic meta-analysis also reported that RPR had almost the same diagnostic performance as APRI and FIB-4 in identifying significant liver fibrosis, while was comparable with APRI and inferior to FIB-4 in staging advanced liver fibrosis and cirrhosis in chronic liver diseases.^[27]^ In non-viral liver diseases, Cengiz et al found that the diagnostic accuracy of RPR was comparable with APRI and FIB-4 for predicting significant liver fibrosis, advanced liver fibrosis, and cirrhosis in NAFLD patients.^[28]^ Wang et al, reported that RPR showed a higher accuracy than APRI and FIB-4 for predicting advanced fibrosis in treatment-naïve PBC patients.^[19]^

Recently, Liu et al assessed the RPR for predicting advanced liver fibrosis in patients with AIH.^[17]^ The study indicated that RPR had the highest accuracy compared to other NITs for predicting advanced liver fibrosis.^[17]^ However, the sample size is relatively simple with only 45 AIH patients included. In addition, this study only investigated the accuracy of RPR in predicting advanced liver fibrosis in AIH patients. Consisted with the study by Liu et al, our study also indicated that RPR could predict advanced liver fibrosis with high accuracy. Furthermore, we evaluated the diagnostic accuracy of RAR for significant liver fibrosis and cirrhosis in AIH patients. Our results showed that PRR could predict significant fibrosis and liver cirrhosis with relative high accuracy.

RPR only contained 2 routine blood routine parameters and the computational formula is relatively simple. Numerous studies reported that RDW was associated with severity of chronic liver diseases.^[29–31]^ A retrospective study by Karagoz et al reported that RDW was significantly increased in CHB patients and can be defined as an independent predictor in liver fibrosis.^[29]^ Our previous study found significantly elevated RDW in patients with CHB related cirrhosis.^[30]^ Kim et al reported that elevated RDW was associated with advanced liver fibrosis in a large cohort of NAFLD.^[31]^ RDW was also demonstrated to be an independent predictor of cirrhosis in AIH.^[16,17]^ Several reasons may interpret the elevation of RDW in AIH patients. Portal hypertension leads to hypersplenism which may increases the destruction of red blood cells.^[17]^ In addition, proinflammatory factor inhibits maturation of red blood cell in AIH patients, which may cause the immature red blood cells into peripheral blood.^[32]^ Moreover, chronic inflammation may impair the iron metabolism, restrain the production of erythropoietin and decrease red blood cell survival which together resulting in the increasing RDW.^[33,34]^ Our previous study also found that the RDW level was positively associated with the severity of liver inflammation in AIH patients.^[35]^ This study revealed that the RDW level was higher in patients with significant liver inflammation than mild inflammation patients, which suggested that RDW may be a promising indicator for reflecting the severity of liver inflammation in AIH patients.^[35]^ PLT is also a well-known independent predictor for liver fibrosis and cirrhosis in chronic liver diseases. The decreased PLT may be caused by hypersplenism and the decreased thrombopoietin production associated with damaged liver cells in liver fibrosis and cirrhosis patients.^[36,37]^

This study has several limitations. First, our study was retrospective and simple size was relatively small. Thus, the diagnosing value of RPR for liver fibrosis in AIH patients remains to be validated in the future studies. Second, we did not compare the predicting performance between RPR and TE since TE was not a routine measure in our clinics. Third, future studies are also required to investigate whether RPR could predict treatment response and long-term outcomes of AIH patients.

In conclusion, the RPR is a more accurate NIT than APRI and FIB-4 to stage liver fibrosis in patients with AIH. The RPR represents a simple and inexpensive alternative NIT to LB for the management of AIH patients in clinic setting.

## Author contributions

Study concept and design: Rui Huang, Chao Wu and Yongfeng Yang; analysis and interpretation of the data: Xiaomin Yan, Yanhong Feng, Jun Chen, Duxian Liu; collection of data: Juan Xia, Lin Li, Weimao Ding; drafting the manuscript: Huali Wang, Jian Wang, and Rui Huang.
